# Beliefs about vaccines and information about coronavirus, COVID-19 and the pandemic. Findings from Italy: Chiara Lorini

**DOI:** 10.1093/eurpub/ckac129.657

**Published:** 2022-10-25

**Authors:** C Lorini, V Velasco, P Zanobini, L Vecchio, G Bonaccorsi

**Affiliations:** 1 Department of Health Science, University of Florence, Florence, Italy; 2 Department of Psychology, University of Milano-Bicocca, Milan, Italy

## Abstract

**Introduction:**

In Italy, COVID-19 vaccination campaign for school personnel started in February 2021 and began mandatory from 15th December 2021 to all the people who work within schools. Here we described Italian school principals’ beliefs about vaccines and its association with feelings about information regarding coronavirus, COVID-19 and the pandemic.

**Methods:**

Data collection started in October 2021 and is ongoing. Beliefs about vaccines were investigated both for COVID-19 vaccination and for vaccination in general.

**Results:**

A total of 726 questionnaires were analyzed so far. The majority feels to be well informed about the coronavirus or the pandemic (75%) and not at all nor a little confused about COVID-19 information (89%). Only 2% disagree/strongly disagree with the statement “vaccination is compatible with my attitudes or religious beliefs”. Beliefs regarding vaccines in general are correlated with those regarding COVID-19 vaccines. When different beliefs are described, we observed a trend towards considering COVID-19 vaccines less safe and effective, but more important to protect themselves and their family than other vaccines. Moreover, beliefs about vaccination in general and regarding COVID-19 are associated with how well they feel informed about the coronavirus or the pandemic, and whether they feel confused about COVID-19 information. In particular: the better they feel informed about the coronavirus and the related pandemic, the higher the perception of vaccines in general and COVID-19 vaccine as important, safe, and effective; the less they feel confused about COVID-19 information.

**Conclusions:**

School principals showed a high level of confidence on vaccines. The association between beliefs in vaccinations and the characteristics of information about COVID-19 supported the effectiveness of Italian vaccination policy and information campaigns.

